# Supplementary material for: Development and temporal validation of a nomogram for predicting ICU 28-day mortality in middle-aged and elderly sepsis patients: An eICU database study
Source: PLoS One. 2025 Jul 21;20(7):e0328701. doi: 10.1371/journal.pone.0328701 (PMC12279146; doi:10.1371/journal.pone.0328701)
Supplement: S1 Table — Data are OR (95% CI) and P value. BMI: Body mass index; MAP: Mean arterial pressure; O2 Sat: Oxygen saturation; PaO2: Partial pressure of arterial oxygen; PaCO2: Partialpressure of arterial carbon dioxide; FiO2: Fraction of inspired oxygen; WBC: White blood cell; RDW: Red cell distribution width; MCHC: Mean corpuscular hemoglobin concentration; BUN: Blood urea nitrogen; ALT: Alanine aminotransferase; AST: Aspartate aminotransferase; PT: Prothrombin time; APTT: Activated partial thromboplastin time; INR: International normalized ratio; GCS: Glasgow coma scale; SOFA: Sequential organ failure assessment; APACHE: Acute physiology and chronic health evaluation; COPD: Chronic obstructive pulmonary disease; CHF: Congestive heart failure; AMI: Acute myocardial infarction; DM: Diabetes mellitus. (DOCX) [file pone.0328701.s001.docx]

| **Exposure** | **OR(95%CI)** | **P value** |
| --- | --- | --- |
| **Demographics** |  |  |
| Age (years) | 1.01 (1.01, 1.02) | 0.0001 |
| Gender |  |  |
| Male | 1.0 |  |
| Female | 1.08 (0.91, 1.28) | 0.3907 |
| BMI (kg/m2) | 0.98 (0.97, 0.99) | 0.0001 |
| Ethnicity |  |  |
| Caucasian | 1.0 |  |
| Other | 1.15 (0.94, 1.40) | 0.1773 |
| Hospital admit source |  |  |
| Emergency Department | 1.0 |  |
| Other | 1.34 (1.12, 1.59) | 0.0010 |
| **Vital signs** |  |  |
| Heart rate (/min) | 1.01 (1.01, 1.01) | <0.0001 |
| Respiratory rate (bpm) | 1.02 (1.01, 1.02) | <0.0001 |
| Temperature | 0.81 (0.76, 0.86) | <0.0001 |
| MAP (mmHg) | 1.00 (1.00, 1.00) | 0.1568 |
| O_2_ Sat (%) | 1.00 (0.98, 1.01) | 0.5321 |
| **Laboratory data** |  |  |
| PH | 0.01 (0.00, 0.04) | <0.0001 |
| PaO_2_ (mmHg) | 1.00 (1.00, 1.00) | 0.0130 |
| PaCO_2_ (mmHg) | 0.99 (0.98, 1.00) | 0.0279 |
| FiO_2_ (%) | 1.00 (1.00, 1.00) | 0.1144 |
| Urine output (24 h, mL) | 1.00 (1.00, 1.00) | <0.0001 |
| Lactate (mmol/L) | 1.25 (1.21, 1.29) | <0.0001 |
| Bicarbonate (mmol/L) | 0.93 (0.92, 0.95) | <0.0001 |
| Base Excess (mmol/L) | 0.94 (0.92, 0.95) | <0.0001 |
| WBC count (cells x 10^9^/L) | 1.01 (1.00, 1.02) | 0.0010 |
| Hemoglobin (g/dL) | 0.99 (0.95, 1.04) | 0.7930 |
| Platelets (cells x 10^9^/L) | 1.00 (1.00, 1.00) | <0.0001 |
| RDW (%) | 1.13 (1.10, 1.17) | <0.0001 |
| MCHC (g/dL) | 0.97 (0.92, 1.03) | 0.3768 |
| Albumin (g/dL) | 0.48 (0.40, 0.58) | <0.0001 |
| Total protein (g/dL) | 0.59 (0.52, 0.66) | <0.0001 |
| Glucose (mg/dl) | 1.00 (1.00, 1.00) | 0.8855 |
| Sodium (mmol/L) | 1.00 (0.99, 1.02) | 0.7393 |
| Serum potassium (mmol/L) | 1.34 (1.21, 1.49) | <0.0001 |
| Calcium (mg/dl) | 0.81 (0.73, 0.90) | <0.0001 |
| Serum creatinine (mg/dL) | 1.10 (1.06, 1.15) | <0.0001 |
| BUN (mg/dL) | 1.01 (1.01, 1.02) | <0.0001 |
| ALT (U/L) | 1.00 (1.00, 1.00) | 0.0003 |
| AST (U/L) | 1.00 (1.00, 1.00) | <0.0001 |
| Total bilirubin (mg/dL) | 1.15 (1.11, 1.19) | <0.0001 |
| Anion gap (mmol/L) | 1.08 (1.06, 1.10) | <0.0001 |
| PT (seconds) | 1.02 (1.01, 1.02) | <0.0001 |
| APTT (seconds) | 1.01 (1.01, 1.02) | <0.0001 |
| INR | 1.14 (1.06, 1.22) | 0.0002 |
| **Site of infection** |  |  |
| Pulmonary | 1.0 |  |
| Other | 0.71 (0.60, 0.84) | <0.0001 |
| **Severity of illness** |  |  |
| GCS score | 0.87 (0.85, 0.89) | <0.0001 |
| SOFA score | 1.26 (1.23, 1.30) | <0.0001 |
| Apache IV score | 1.04 (1.03, 1.04) | <0.0001 |
| Acute Physiology Score III | 1.04 (1.03, 1.04) | <0.0001 |
| **Past medical history** |  |  |
| COPD |  |  |
| No | 1.0 |  |
| Yes | 0.81 (0.59, 1.09) | 0.1669 |
| CHF |  |  |
| No | 1.0 |  |
| Yes | 1.40 (1.08, 1.82) | 0.0125 |
| AMI |  |  |
| No | 1.0 |  |
| Yes | 1.83 (1.22, 2.75) | 0.0033 |
| DM |  |  |
| No | 1.0 |  |
| Yes | 0.72 (0.56, 0.94) | 0.0137 |
| Pneumonia |  |  |
| No | 1.0 |  |
| Yes | 1.46 (1.23, 1.74) | <0.0001 |
| Rhythm |  |  |
| No | 1.0 |  |
| Yes | 1.80 (1.48, 2.19) | <0.0001 |
| **Intervention** |  |  |
| Mechanical ventilation |  |  |
| No | 1.0 |  |
| Yes | 2.61 (2.19, 3.10) | <0.0001 |
| Dialysis |  |  |
| No | 1.0 |  |
| Yes | 1.00 (0.69, 1.47) | 0.9912 |
| Vasopressor use (1st 24 h) |  |  |
| No | 1.0 |  |
| Yes | 1.88 (0.92, 3.85) | 0.0845 |
