# Supplementary material for: Development and temporal validation of a nomogram for predicting ICU 28-day mortality in middle-aged and elderly sepsis patients: An eICU database study
Source: PLoS One. 2025 Jul 21;20(7):e0328701. doi: 10.1371/journal.pone.0328701 (PMC12279146; doi:10.1371/journal.pone.0328701)
Supplement: S2 Table — Data are variance inflation factor (VIF) values. NA indicates the variable was removed from the model at that step. Variables eliminated (VIF > 5): Bicarbonate (VIF = 17.0), Base Excess (VIF = 18.2), ALT (VIF = 9.9), PT (VIF = 66.2), APACHE IV score (VIF = 69.6), and Acute Physiology Score III (VIF = 73.0). BMI: Body mass index; GCS: Glasgow coma scale; PaCO2: Partial pressure of arterial carbon dioxide; FiO2: Fraction of inspired oxygen; WBC: White blood cell; RDW: Red cell distribution width; BUN: Blood urea nitrogen; ALT: Alanine aminotransferase; AST: Aspartate aminotransferase; PT: Prothrombin time; APTT: Activated partial thromboplastin time; INR: International normalized ratio; SOFA: Sequential organ failure assessment; APACHE: Acute physiology and chronic health evaluation; CHF: Congestive heart failure; AMI: Acute myocardial infarction; DM: Diabetes mellitus. (DOCX) [file pone.0328701.s002.docx]

|  | **Step 1** | **Step 2** | **Step 3** | **Step 4** | **Step 5** | **Step 6** | **Step 7** |
| --- | --- | --- | --- | --- | --- | --- | --- |
| **Age** | 4 | 2.5 | 2.3 | 2 | 2 | 1.8 | 1.7 |
| **BMI** | 1.9 | 1.9 | 1.9 | 1.8 | 1.8 | 1.6 | 1.5 |
| **Hospital admit source** | 1.9 | 1.9 | 1.8 | 1.5 | 1.5 | 1.5 | 1.4 |
| **Heart rate** | 2.2 | 2.1 | 2 | 1.9 | 1.9 | 1.4 | 1.4 |
| **Respiratory rate** | 1.9 | 1.9 | 1.9 | 1.9 | 1.8 | 1.5 | 1.5 |
| **Temperature** | 2.1 | 2.1 | 2 | 1.7 | 1.7 | 1.5 | 1.4 |
| **GCS score** | 4.5 | 4.4 | 4.1 | 3.6 | 3.6 | 1.9 | 1.9 |
| **PH** | 4.7 | 4.7 | 4.6 | 2.8 | 2.7 | 2.4 | 1.9 |
| **PaCO_2_** | 4.4 | 4.3 | 4 | 3.5 | 3.5 | 3.4 | 1.9 |
| **FiO_2_** | 1.9 | 1.8 | 1.8 | 1.6 | 1.6 | 1.5 | 1.5 |
| **Lactate** | 2.7 | 2.7 | 2.6 | 2.6 | 2.5 | 2.2 | 2.1 |
| **Bicarbonate** | 17 | 16.6 | 16.2 | 6.4 | 6.1 | 6.3 | NA |
| **Base Excess** | 18.2 | 18 | 18.1 | NA | NA | NA | NA |
| **WBC count** | 2 | 2 | 2 | 1.7 | 1.7 | 1.5 | 1.4 |
| **Platelets** | 1.9 | 1.8 | 1.7 | 1.7 | 1.6 | 1.5 | 1.4 |
| **RDW** | 1.8 | 1.8 | 1.8 | 1.6 | 1.6 | 1.7 | 1.6 |
| **Albumin** | 2.3 | 2.3 | 2.2 | 2.2 | 2.2 | 2 | 1.9 |
| **Total protein** | 2 | 2 | 2 | 1.8 | 1.8 | 1.9 | 1.6 |
| **Serum potassium** | 2 | 2 | 2 | 1.9 | 1.8 | 1.7 | 1.7 |
| **Calcium** | 3.2 | 3.2 | 2.9 | 2.3 | 2.3 | 2.1 | 2 |
| **Serum creatinine** | 4.7 | 4.6 | 4.3 | 3.9 | 3.9 | 3.1 | 3.2 |
| **BUN** | 4.7 | 4.7 | 4.5 | 3.8 | 3.8 | 2.9 | 2.8 |
| **ALT** | 9.9 | 9.9 | 9.7 | 8.2 | NA | NA | NA |
| **AST** | 9.2 | 9.2 | 9.3 | 8.1 | 1.8 | 1.7 | 1.7 |
| **Total bilirubin** | 2.2 | 2.1 | 1.7 | 1.6 | 1.6 | 1.5 | 1.4 |
| **Anion gap** | 5.3 | 5.2 | 5.2 | 4.1 | 4 | 3.7 | 2.7 |
| **PT** | 66.2 | 66.2 | NA | NA | NA | NA | NA |
| **APTT** | 4.4 | 4.2 | 3.9 | 3 | 3 | 1.9 | 1.9 |
| **INR** | 65.2 | 65.1 | 3.7 | 2.8 | 2.8 | 1.9 | 1.8 |
| **SOFA score** | 4.5 | 4.3 | 4.2 | 3.3 | 3.2 | 3 | 2.8 |
| **Apache IV score** | 69.6 | 8.8 | 8.1 | 7 | 6.9 | NA | NA |
| **Acute Physiology Score III** | 73 | NA | NA | NA | NA | NA | NA |
| **Mechanical ventilation** | 1.9 | 1.9 | 1.7 | 1.7 | 1.7 | 1.6 | 1.4 |
| **Urine output** | 1.7 | 1.7 | 1.7 | 1.7 | 1.7 | 1.5 | 1.3 |
| **CHF** | 2 | 2 | 2 | 1.7 | 1.6 | 1.5 | 1.4 |
| **AMI** | 1.7 | 1.7 | 1.7 | 1.6 | 1.6 | 1.5 | 1.5 |
| **DM** | 1.8 | 1.8 | 1.7 | 1.5 | 1.4 | 1.4 | 1.3 |
| **Pneumonia** | 1.8 | 1.8 | 1.8 | 1.7 | 1.7 | 1.7 | 1.7 |
| **Rhythm** | 1.7 | 1.7 | 1.7 | 1.6 | 1.5 | 1.4 | 1.4 |
| **Site of infection** | 2.2 | 2.1 | 2 | 1.9 | 1.9 | 2 | 2 |
