# Supplementary material for: Development and temporal validation of a nomogram for predicting ICU 28-day mortality in middle-aged and elderly sepsis patients: An eICU database study
Source: PLoS One. 2025 Jul 21;20(7):e0328701. doi: 10.1371/journal.pone.0328701 (PMC12279146; doi:10.1371/journal.pone.0328701)
Supplement: S3 Table — SET params as: n_trees = 500, split_features = 6, total_features = 34, sampling = swor (without replacement), resample_size = 4043, splitting_rule = gini random, random_split_points = 10, class_imbalance_ratio = 10.01. The table shows all important variables ranked by total importance score in the random forest model. Total importance represents the overall predictive power of each variable. Positive impact indicates the variable’s contribution to predicting mortality when its value increases, while negative impact indicates its contribution to predicting survival. Analysis was performed using the training cohort (patients discharged in 2014, n = 6,397). SOFA: Sequential organ failure assessment; WBC: White blood cell; RDW: Red cell distribution width; PaCO2: Partial pressure of arterial carbon dioxide; GCS: Glasgow coma scale; FiO2: Fraction of inspired oxygen; BUN: Blood urea nitrogen; INR: International normalized ratio; AST: Aspartate aminotransferase; APTT: Activated partial thromboplastin time; BMI: Body mass index; AMI: Acute myocardial infarction; CHF: Congestive heart failure; DM: Diabetes mellitus. (DOCX) [file pone.0328701.s003.docx]

| **Variable** | **All** | **Positive Impact** | **Negative Impact** |
| --- | --- | --- | --- |
| Lactate | 0.060 | 0.188 | -0.118 |
| pH | 0.050 | 0.163 | -0.157 |
| SOFA score | 0.046 | 0.146 | -0.115 |
| Calcium | 0.037 | 0.127 | -0.181 |
| WBC count | 0.035 | 0.121 | -0.165 |
| Serum potassium | 0.031 | 0.103 | -0.121 |
| Temperature | 0.031 | 0.104 | -0.131 |
| Total bilirubin | 0.030 | 0.105 | -0.149 |
| RDW | 0.029 | 0.097 | -0.114 |
| PaCO2 | 0.029 | 0.096 | -0.104 |
| GCS score | 0.028 | 0.094 | -0.110 |
| Heart rate | 0.028 | 0.094 | -0.109 |
| Total protein | 0.028 | 0.094 | -0.121 |
| Platelets | 0.027 | 0.092 | -0.126 |
| FiO2 | 0.025 | 0.082 | -0.068 |
| Albumin | 0.022 | 0.073 | -0.077 |
| BUN | 0.020 | 0.066 | -0.066 |
| Anion gap | 0.017 | 0.059 | -0.080 |
| INR | 0.017 | 0.059 | -0.083 |
| AST | 0.017 | 0.056 | -0.055 |
| APTT | 0.016 | 0.054 | -0.068 |
| BMI | 0.015 | 0.054 | -0.086 |
| AMI | 0.015 | 0.048 | -0.049 |
| Urine output | 0.014 | 0.044 | -0.018 |
| Respiratory rate | 0.010 | 0.033 | -0.029 |
| Age | 0.009 | 0.029 | -0.029 |
| Mechanical ventilation | 0.009 | 0.028 | -0.019 |
| Serum creatinine | 0.009 | 0.029 | -0.037 |
| Cardiac rhythm | 0.008 | 0.026 | -0.023 |
| CHF | 0.006 | 0.021 | -0.023 |
| Pneumonia | 0.003 | 0.011 | -0.012 |
| Site of infection | 0.002 | 0.006 | -0.004 |
| DM | 0.000 | 0.002 | -0.002 |
| Hospital admission source | 0.000 | 0.000 | 0.000 |
