# Supplementary figures and images for: Development and temporal validation of a nomogram for predicting ICU 28-day mortality in middle-aged and elderly sepsis patients: An eICU database study
Source: PLoS One. 2025 Jul 21;20(7):e0328701. doi: 10.1371/journal.pone.0328701 (PMC12279146; doi:10.1371/journal.pone.0328701)

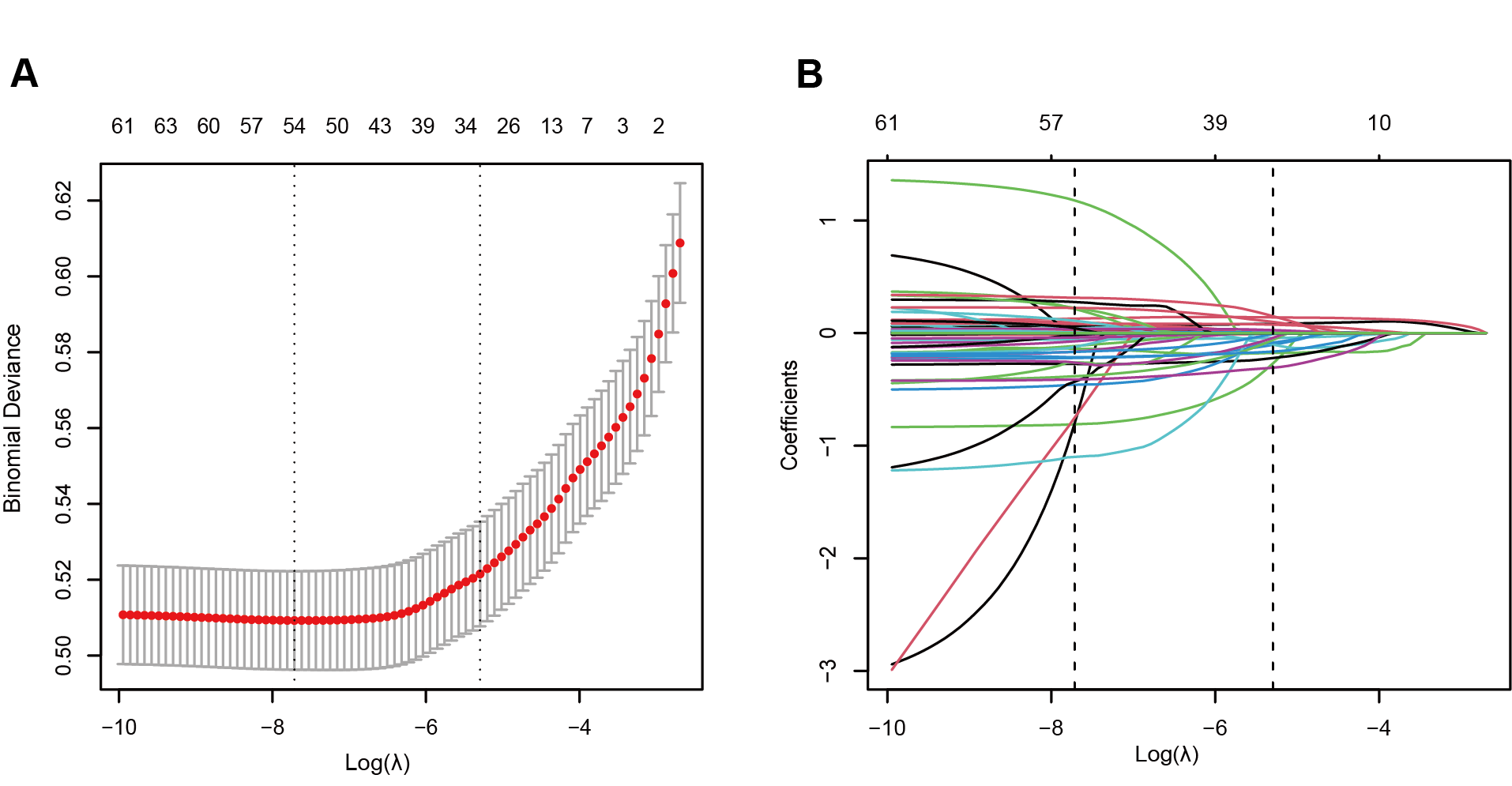

Supplement: S1 Fig — (B) Coefficient path plot showing variable selection with log(lambda) values. (PNG) [file pone.0328701.s004.png]

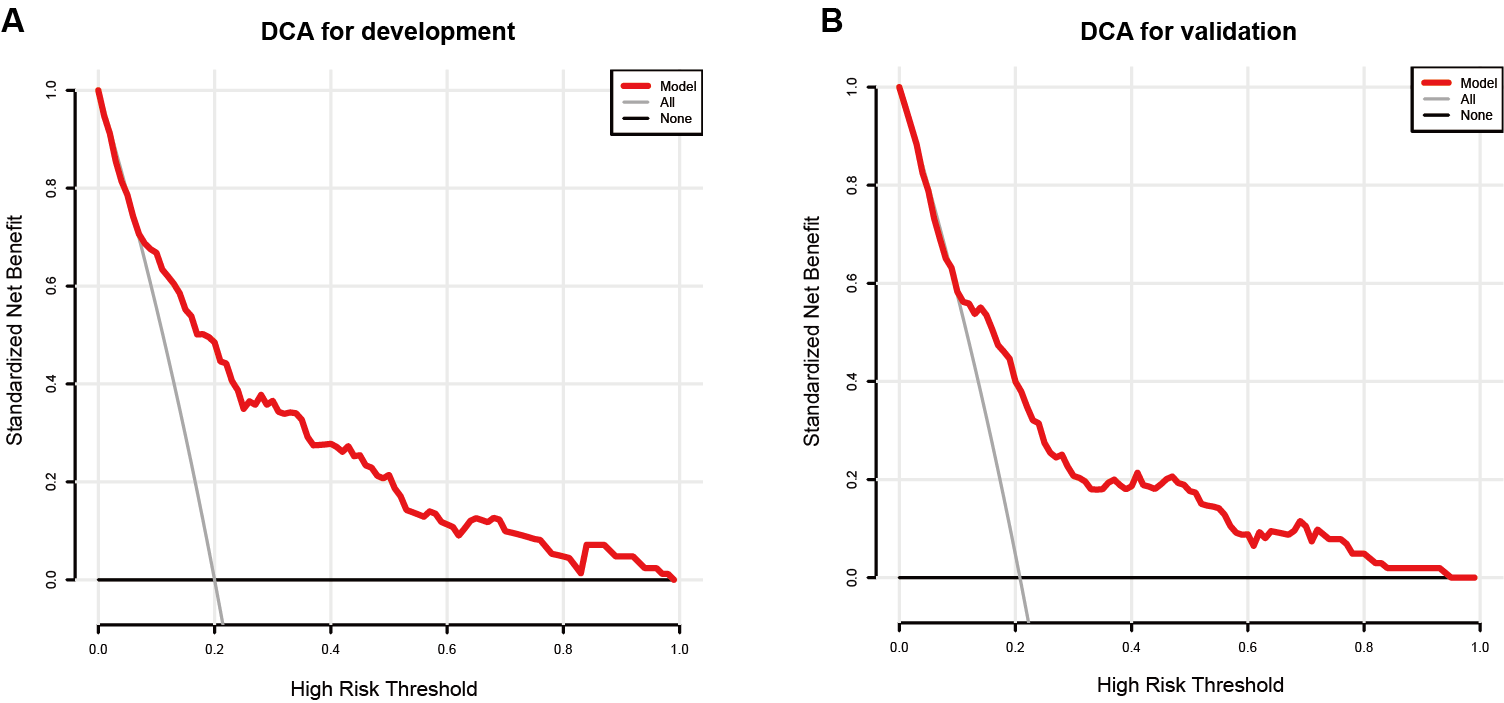

Supplement: S2 Fig — (A) Development cohort. (B) Validation cohort. The grey line represents the net benefit of treating all patients, the black line represents treating no patients, and the red line represents the net benefit of the prediction model at different threshold probabilities. ICU, Intensive Care Unit; DCA, Decision Curve Analysis. (PNG) [file pone.0328701.s007.png]
