# Supplementary material for: Development and temporal validation of a nomogram for predicting ICU 28-day mortality in middle-aged and elderly sepsis patients: An eICU database study
Source: PLoS One. 2025 Jul 21;20(7):e0328701. doi: 10.1371/journal.pone.0328701 (PMC12279146; doi:10.1371/journal.pone.0328701)
Supplement: S4 Table — Data are expressed as the mean±SD, median (interquartile range), or percentage. BMI: Body mass index; MAP: Mean arterial pressure; O2 Sat: Oxygen saturation; PaO2: Partial pressure of arterial oxygen; PaCO2: Partial pressure of arterial carbon dioxide; FiO2: Fraction of inspired oxygen; WBC White blood cell; RDW: Red cell distribution width; MCHC: Mean corpuscular hemoglobin concentration; BUN: Blood urea nitrogen; ALT: Alanine aminotransferase; AST: Aspartate aminotransferase; PT: Prothrombin time; APTT: Activated partial thromboplastin time; INR: International normalized ratio; GCS: Glasgow coma scale; SOFA: Sequential organ failure assessment; APACHE: Acute physiology and chronic health evaluation; COPD: Chronic obstructive pulmonary disease; CHF: Congestive heart failure; AMI: Acute myocardial infarction; DM: Diabetes mellitus. (DOCX) [file pone.0328701.s005.docx]

|  | **Complete data**  **（N=911）** | **Missing data**  **（N=12806）** | **P value** |
| --- | --- | --- | --- |
| **Demographics** |  |  |  |
| Age (years) | 67.5 ± 11.9 | 69.7 ± 12.1 | <0.001 |
| BMI (kg/m^2^) | 29.4 ± 9.1 | 28.9 ± 8.9 | 0.084 |
| Gender |  |  | 0.036 |
| Male | 414 (45.4%) | 6277 (49.0%) |  |
| Female | 497 (54.6%) | 6524 (51.0%) |  |
| Ethnicity |  |  | 0.780 |
| Caucasian | 713 (78.3%) | 10073 (78.7%) |  |
| Other | 198 (21.7%) | 2733 (21.3%) |  |
| Hospital admit source |  |  | <0.001 |
| Emergency Department | 132 (31.4%) | 3027 (50.6%) |  |
| Other | 288 (68.6%) | 2950 (49.4%) |  |
| **Vital signs** |  |  |  |
| Heart rate (/min) | 117.6 ± 29.3 | 110.3 ± 28.7 | <0.001 |
| Respiratory rate (bpm) | 32.2 ± 14.8 | 29.8 ± 14.2 | <0.001 |
| Temperature (**℃**) | 36.5 ± 1.4 | 36.6 ± 1.2 | 0.125 |
| MAP (mmHg) | 55.0 (46.0-129.0) | 56.0 (47.0-108.0) | 0.698 |
| O2 Sat (%) | 91.0 (0.6-97.0) | 94.2 (2.8-98.0) | <0.001 |
| **Laboratory data** |  |  |  |
| PH | 24.9 (7.3-39.6) | 20.7 (7.4-38.4) | 0.863 |
| paO2 (mm Hg) | 31.2 (20.2-91.4) | 37.5 (21.4-90.0) | 0.040 |
| PaCO_2_ (mmHg) | 44.0 (33.8-65.0) | 40.0 (30.0-55.0) | <0.001 |
| FiO2 (%) | 50.0 (28.0-100.0) | 40.0 (21.0-65.0) | <0.001 |
| Urine output (24 h, mL) | 1047.8 (413.7-2024.5) | 1242.3 (616.9-2196.2) | <0.001 |
| Lactate (mmol/L) | 2.0 (1.2-3.4) | 1.8 (1.2-2.9) | <0.001 |
| Bicarbonate (mmol/L) | 21.6 ± 6.1 | 22.4 ± 5.5 | <0.001 |
| Base Excess (mmol/L) | 70.2 (2.0-104.0) | 66.0 (0.2-101.0) | 0.031 |
| WBC count (cells x 10^9^/L) | 80.0 (15.3-93.0) | 78.0 (13.8-91.7) | 0.047 |
| Hemoglobin (g/dL) | 13.7 ± 3.9 | 13.3 ± 3.7 | 0.005 |
| Platelets (cells x 10^9^/L) | 4.7 (3.5-168.0) | 4.7 (3.4-173.0) | 0.743 |
| RDW (%) | 25.2 ± 9.9 | 24.8 ± 9.2 | 0.310 |
| MCHC (g/dL) | 31.1 ± 2.8 | 31.1 ± 2.7 | 0.723 |
| Albumin (g/dL) | 2.5 ± 0.6 | 2.5 ± 0.6 | 0.358 |
| Total protein (g/dL) | 5.6 ± 0.9 | 5.7 ± 0.9 | <0.001 |
| Glucose (mg/dl) | 134.0 (103.5-183.5) | 128.0 (102.0-169.0) | 0.004 |
| Sodium (mmol/L) | 138.4 ± 6.3 | 138.4 ± 6.3 | 0.931 |
| Serum potassium (mmol/L) | 4.2 ± 0.8 | 4.1 ± 0.8 | <0.001 |
| Calcium (mg/dl) | 7.8 ± 0.9 | 8.0 ± 0.9 | <0.001 |
| Serum creatinine (mg/dL) | 2.0 ± 1.6 | 2.0 ± 1.8 | 0.254 |
| BUN (mg/dL) | 32.0 (20.0-51.0) | 29.0 (18.0-46.0) | <0.001 |
| ALT (U/L) | 28.0 (17.0-64.0) | 27.0 (16.0-54.0) | 0.052 |
| AST (U/L) | 41.0 (23.0-93.5) | 36.0 (21.0-76.0) | <0.001 |
| Total bilirubin (mg/dL) | 0.7 (0.4-1.3) | 0.7 (0.4-1.2) | 0.527 |
| Anion gap(mmol/L) | 12.1 ± 5.0 | 12.0 ± 4.9 | 0.651 |
| PT (seconds) | 23.5 (16.1-33.8) | 25.2 (16.4-36.0) | 0.059 |
| APTT (seconds) | 124.0 (42.0-215.0) | 156.0 (72.0-233.0) | <0.001 |
| INR | 5.2 (1.4-17.1) | 11.5 (1.5-17.7) | 0.037 |
| **Site of infection** |  |  | <0.001 |
| Pulmonary | 208 (49.5%) | 2258 (37.8%) |  |
| Other | 212 (50.5%) | 3719 (62.2%) |  |
| **Severity of illness** |  |  |  |
| GCS score | 10.7 ± 3.9 | 12.7 ± 3.3 | <0.001 |
| SOFA score | 6.3 ± 3.4 | 4.2 ± 2.8 | <0.001 |
| Apache IV score | 96.6 ± 29.8 | 69.6 ± 23.6 | <0.001 |
| Acute Physiology Score III | 82.4 ± 28.9 | 54.4 ± 22.4 | <0.001 |
| **Past medical history** |  |  |  |
| COPD |  |  | 0.110 |
| No | 810 (88.9%) | 11593 (90.5%) |  |
| Yes | 101 (11.1%) | 1213 (9.5%) |  |
| CHF |  |  | 0.053 |
| No | 812 (89.1%) | 11659 (91.0%) |  |
| Yes | 99 (10.9%) | 1147 (9.0%) |  |
| AMI |  |  | 0.004 |
| No | 863 (94.7%) | 12368 (96.6%) |  |
| Yes | 48 (5.3%) | 438 (3.4%) |  |
| DM |  |  | 0.987 |
| No | 780 (85.6%) | 10967 (85.6%) |  |
| Yes | 131 (14.4%) | 1839 (14.4%) |  |
| Pneumonia |  |  | <0.001 |
| No | 499 (54.8%) | 8513 (66.5%) |  |
| Yes | 412 (45.2%) | 4293 (33.5%) |  |
| Rhythm |  |  | <0.001 |
| No | 689 (75.6%) | 10655 (83.2%) |  |
| Yes | 222 (24.4%) | 2151 (16.8%) |  |
| **Intervention** |  |  |  |
| Mechanical ventilation |  |  | <0.001 |
| No | 401 (44.0%) | 9207 (73.1%) |  |
| Yes | 510 (56.0%) | 3386 (26.9%) |  |
| Dialysis |  |  | <0.001 |
| 0 | 888 (97.5%) | 11899 (94.5%) |  |
| 1 | 23 (2.5%) | 694 (5.5%) |  |
| Vasopressor use (1st 24 h) |  |  | 0.007 |
| No | 911 (100.0%) | 12457 (99.2%) |  |
| Yes | 0 (0.0%) | 98 (0.8%) |  |
