# Supplementary material for: Development and temporal validation of a nomogram for predicting ICU 28-day mortality in middle-aged and elderly sepsis patients: An eICU database study
Source: PLoS One. 2025 Jul 21;20(7):e0328701. doi: 10.1371/journal.pone.0328701 (PMC12279146; doi:10.1371/journal.pone.0328701)
Supplement: S5 Table — Data are expressed as the mean±SD, median (interquartile range), or percentage. BMI: Body mass index; MAP: Mean arterial pressure; O2 Sat: Oxygen saturation; PaO2: Partial pressure of arterial oxygen; PaCO2: Partial pressure of arterial carbon dioxide; FiO2: Fraction of inspired oxygen; WBC White blood cell; RDW: Red cell distribution width; MCHC: Mean corpuscular hemoglobin concentration; BUN: Blood urea nitrogen; ALT: Alanine aminotransferase; AST: Aspartate aminotransferase; PT: Prothrombin time; APTT: Activated partial thromboplastin time; INR: International normalized ratio; GCS: Glasgow coma scale; SOFA: Sequential organ failure assessment; APACHE: Acute physiology and chronic health evaluation; COPD: Chronic obstructive pulmonary disease; CHF: Congestive heart failure; AMI: Acute myocardial infarction; DM: Diabetes mellitus. (DOCX) [file pone.0328701.s006.docx]

|  | **Training cohort**  **(N=6397)** | **Validation cohort**  **(N=7320)** | **P value** |
| --- | --- | --- | --- |
| **Demographics** |  |  |  |
| Age (years) | 69.5 ± 12.3 | 69.6 ± 12.0 | 0.550 |
| BMI (kg/m^2^) | 29.0 ± 9.0 | 28.9 ± 8.8 | 0.540 |
| Gender |  |  | 0.206 |
| Male | 3157 (49.4%) | 3534 (48.3%) |  |
| Female | 3237 (50.6%) | 3784 (51.7%) |  |
| Ethnicity |  |  | <0.001 |
| Caucasian | 4933 (77.1%) | 5853 (80.0%) |  |
| Other | 1464 (22.9%) | 1467 (20.0%) |  |
| Hospital admit source |  |  | 0.249 |
| Emergency Department | 3159 (49.4%) | 3687 (50.4%) |  |
| Other | 3238 (50.6%) | 3633 (49.6%) |  |
| **Vital signs** |  |  |  |
| Heart rate (/min) | 110.8 ± 28.1 | 110.8 ± 29.4 | 0.993 |
| Respiratory rate (bpm) | 29.4 ± 14.4 | 30.5 ± 14.1 | <0.001 |
| Temperature (**℃**) | 36.5 ± 1.3 | 36.6 ± 1.1 | 0.467 |
| MAP (mmHg) | 55.0 (46.0-110.0) | 56.0 (47.0-111.0) | 0.023 |
| O_2_ Sat (%) | 93.6 ± 9.3 | 94.3 ± 8.1 | 0.004 |
| **Laboratory data** |  |  |  |
| PH | 7.3 ± 0.1 | 7.3 ± 0.1 | 0.611 |
| PaO_2_ (mmHg) | 90.0 (70.0-131.0) | 91.0 (72.0-132.0) | 0.121 |
| PaCO_2_ (mmHg) | 40.9 ± 14.6 | 41.1 ± 14.8 | 0.441 |
| FiO_2_ (%) | 44.5 (29.0-80.0) | 45.0 (29.0-80.0) | 0.896 |
| Urine output (24 h, mL) | 1183.7 (586.1-2137.4) | 1234.8 (580.6-2201.2) | 0.476 |
| Lactate (mmol/L) | 1.8 (1.1-2.9) | 1.9 (1.2-3.1) | <0.001 |
| Bicarbonate (mmol/L) | 22.5 ± 5.5 | 22.3 ± 5.5 | 0.056 |
| Base Excess (mmol/L) | -2.0 (-7.0-2.0) | -2.8 (-7.1-2.0) | 0.154 |
| WBC count (cells x 10^9^/L) | 13.4 (9.0-19.4) | 13.2 (8.7-19.1) | 0.067 |
| Hemoglobin (g/dL) | 10.4 ± 2.1 | 10.4 ± 2.1 | 0.826 |
| Platelets (cells x 10^9^/L) | 181.0 (124.0-256.0) | 182.0 (125.0-254.0) | 0.905 |
| RDW (%) | 16.1 ± 2.6 | 16.1 ± 2.6 | 0.724 |
| MCHC (g/dL) | 32.7 ± 1.5 | 32.5 ± 1.5 | <0.001 |
| Albumin (g/dL) | 2.5 ± 0.6 | 2.5 ± 0.6 | <0.001 |
| Total protein (g/dL) | 5.7 ± 0.9 | 5.7 ± 0.9 | 0.130 |
| Glucose (mg/dl) | 128.0 (102.0-170.0) | 128.5 (102.8-171.0) | 0.482 |
| Sodium (mmol/L) | 138.3 ± 6.4 | 138.4 ± 6.3 | 0.629 |
| Serum potassium (mmol/L) | 4.1 ± 0.8 | 4.1 ± 0.8 | 0.085 |
| Calcium (mg/dl) | 8.0 ± 0.9 | 8.0 ± 0.9 | 0.663 |
| Serum creatinine (mg/dL) | 2.0 ± 1.8 | 2.0 ± 1.8 | 0.423 |
| BUN (mg/dL) | 30.0 (18.0-48.0) | 29.0 (18.0-46.0) | 0.097 |
| ALT (U/L) | 28.0 (17.0-54.0) | 27.0 (16.0-55.0) | 0.406 |
| AST (U/L) | 36.0 (21.0-77.0) | 36.0 (21.0-78.0) | 0.631 |
| Total bilirubin (mg/dL) | 0.7 (0.4-1.2) | 0.7 (0.4-1.3) | 0.108 |
| Anion gap (mmol/L) | 12.1 ± 4.8 | 12.0 ± 4.9 | 0.492 |
| PT (seconds) | 17.2 (14.8-22.8) | 17.4 (14.8-23.7) | 0.294 |
| APTT (seconds) | 35.2 (30.6-43.1) | 35.6 (31.0-43.2) | 0.647 |
| INR | 1.5 (1.2-2.1) | 1.5 (1.2-2.1) | 0.351 |
| **Site of infection** |  |  | 0.164 |
| Pulmonary | 2466 (38.5%) | 2907 (39.7%) |  |
| Other | 3931 (61.5%) | 4413 (60.3%) |  |
| **Severity of illness** |  |  |  |
| GCS score | 12.5 ± 3.4 | 12.6 ± 3.3 | 0.108 |
| SOFA score | 4.0 (2.0-6.0) | 4.0 (2.0-6.0) | 0.022 |
| Apache IV score | 71.9 ± 24.9 | 71.1 ± 25.1 | 0.075 |
| Acute Physiology Score III | 56.8 ± 23.9 | 56.0 ± 24.0 | 0.060 |
| **Past medical history** |  |  |  |
| COPD |  |  | 0.061 |
| No | 5752 (89.9%) | 6651 (90.9%) |  |
| Yes | 645 (10.1%) | 669 (9.1%) |  |
| CHF |  |  | 0.343 |
| No | 5800 (90.7%) | 6671 (91.1%) |  |
| Yes | 597 (9.3%) | 649 (8.9%) |  |
| AMI |  |  | <0.001 |
| No | 6206 (97.0%) | 7025 (96.0%) |  |
| Yes | 191 (3.0%) | 295 (4.0%) |  |
| DM |  |  | <0.001 |
| No | 5399 (84.4%) | 6348 (86.7%) |  |
| Yes | 998 (15.6%) | 972 (13.3%) |  |
| Pneumonia |  |  | 0.041 |
| No | 4146 (64.8%) | 4866 (66.5%) |  |
| Yes | 2251 (35.2%) | 2454 (33.5%) |  |
| Rhythm |  |  | 0.026 |
| No | 5241 (81.9%) | 6103 (83.4%) |  |
| Yes | 1156 (18.1%) | 1217 (16.6%) |  |
| **Intervention** |  |  |  |
| Mechanical ventilation |  |  | 0.884 |
| No | 4475 (71.1%) | 5133 (71.2%) |  |
| Yes | 1820 (28.9%) | 2076 (28.8%) |  |
| Dialysis |  |  | 0.832 |
| No | 5958 (94.6%) | 6829 (94.7%) |  |
| Yes | 337 (5.4%) | 380 (5.3%) |  |
| Vasopressor use (1st 24 h) |  |  | 0.021 |
| No | 6219 (99.1%) | 7149 (99.4%) |  |
| Yes | 57 (0.9%) | 41 (0.6%) |  |
